# Efficacy of Prehospital Spine and Limb Immobilization in Multiple Trauma Patients

**DOI:** 10.5812/traumamon.16610

**Published:** 2014-08-01

**Authors:** Mohsen Adib-Hajbaghery, Farzaneh Maghaminejad, Mahdi Rajabi

**Affiliations:** 1 Trauma Nursing Research Center, Kashan University of Medical Sciences, Kashan, IR Iran; 2 Department of Medical Surgical Nursing, Faculty of Nursing and Midwifery, Kashan University of Medical Sciences, Kashan, IR Iran; 3 Department of Anesthesiology, Kashan University of Medical Sciences, Kashan, IR Iran

**Keywords:** Epidemiology, Healthcare Quality, Emergency Care, Prehospital, Immobilization, Multiple Trauma

## Abstract

**Background:**

Injuries are a major cause of mortality and disability worldwide and are estimated to become the third leading cause of death by 2020. Most traffic deaths occur during the prehospital phase; consequently, prehospital trauma care has received considerable attention during the past decade. However, there is no study on the prehospital immobilization of spine and limbs in patients with multiple trauma in Iran.

**Objectives:**

This study aimed to investigate the epidemiology of trauma and the quality of limb and spine immobilization in patients with multiple trauma transferred to Shahid Beheshti Medical Center via emergency medical services (EMS).

**Patients and Methods:**

This cross-sectional study was conducted in 2013. The study population consisted of all patients with multiple trauma who had been transferred by EMS to the Central Trauma Department of the Shahid Beheshti Medical Center, Kashan, Iran. The study used a checklist and we recruited a convenience sample of 400 patients with multiple trauma. Data were described by using frequency tables, central tendency measures, and variability indices. Moreover, we analyzed data using SPSS.

**Results:**

The study sample consisted of 301 (75.2%) males and 99 (24.8%) females. The most common mechanism of trauma was traffic injuries (87.25%). Motorcyclists constituted 52.25% of the road traffic injuries victims. Overall, the quality of immobilization was at an undesirable level in 95.8% of patients with spine and limbs injuries. A significant association was observed between the quality of spine and limbs immobilization and the EMS workers’ education level (P = 0.005).

**Conclusions:**

The quality of spine and limb immobilizations was undesirable in more than 90% of cases. Due to the importance of good spine and limb immobilization in patients with multiple trauma, prehospital EMS technicians should be retrained for proper immobilization in patients suspected of spine or limb injuries. Developing evidence-based protocols and strengthening the regulatory and supervisory system to improve quality of prehospital emergency care in patients with multiple trauma is recommended.

## 1. Background

Trauma is a major cause of mortality and disability worldwide (1) with more than five million deaths each year (2). In Europe, nearly 800000 people die from injuries every year (3). The death rate due to road traffic injuries (RTIs) in Iran was about 31 per 100000 populations in 2011 (4). Most of the victims were 20 to 30 years of age (5).

Patients with significant blunt trauma are assumed by prehospital emergency care staff to have potential spinal injuries (6), because about 2% of all patients with blunt trauma may sustain spinal cord injury (SCI) (7). Each year in the United States, approximately 11000 to 12000 individuals sustain SCI from RTIs, sport-related injuries, and direct trauma (8). Patients with SCI are at risk of neurologic deterioration due to secondary injury to the spinal cord (8). Approximately, 20% of these patients die before admission to the hospital (9). Therefore, all trauma patients with a cervical SCI or those with a mechanism of injury that has the potential to cause cervical spine injury, should be immobilized at the scene and during transportation by using one of several available methods (10). Appropriate immobilization may reduce the chance of permanent neurological deficit or additional loss of neurological function (11).

In a four-year prospective study, Domeier et al. evaluated the performance of EMS staff in prehospital spine immobilization. Their results showed that from 13357 patients, 415 individuals had confirmed spinal cord or cervical injuries. However, spine immobilization had not been performed for 8% of patients who needed this procedure while it was performed for 12% of patients who did not (12). Ahmadi Amoli et al. also studied the efficacy of prehospital care in patients with trauma and reported that cervical spine collar and long spine backboards were not used in 80% of injured individuals who needed them (13). In addition, in a systematic review, Ahn et al. evaluated the optimal type and duration of prehospital spine immobilization in patients with acute SCI and the role of prehospital care providers in cervical spine immobilization (7). However, their report did not concern the quality of prehospital spine immobilization in the reviewed studies.

These studies indicate that despite vital importance of immobilization of limbs and spine such issues are sometimes neglected in emergency situations. However, few studies are available on the quality of limb and spine immobilization in patients with multiple trauma, especially in eastern countries. 

## 2. Objectives

This study aimed to investigate the epidemiology of trauma and the quality of limb and spine immobilization in patients with multiple trauma transferred by EMS to the Shahid Beheshti Medical Center. 

## 3. Patients and Methods

This cross-sectional study was conducted from April through September 2013. In order to calculate the sample size, information about the patients with multiple trauma in the same period of the preceding year was obtained from the archives of the emergency department at Shahid Beheshti Medical Center and the records in the prehospital EMS in Kashan, Iran. Based on the recorded data, 350 patients with multiple trauma had been recorded in the same period during 2012. Then the number of samples was estimated to be about 350 patients; however, 400 patients with multiple traumas were referred during the determined period. The study population consisted of all patients with multiple traumas who had been transferred by EMS to Shahid Beheshti Medical Center, which is the main trauma center in Kashan and is affiliated to Kashan University of Medical Sciences, Kashan, Iran. The inclusion criteria were having multiple trauma, alive at admission, and transferred to the trauma center by EMS. All the patients with inclusion criteria were recruited consecutively.

The study instrument consisted of three parts namely a demographic questionnaire, the 6-item Trauma Assessment Questionnaire (TAQ), and the nine-item Spine and Limb Immobilization Quality Assessment Scale (SLIQAS) designed by the researchers. 

The demographic questionnaire consisted of four questions regarding patient age, gender, occupation, and education level. The TAQ included questions concerning the occurrence date of trauma, the type of trauma (blunt, penetrating, or both), the mechanism of trauma (RTIs, fall, street fight, and falling objects), the type of RTI (pedestrian, bicycle, motorcycle, and car), the time of trauma (day or night), and the place of trauma (home, workplace, urban streets, and country roads). 

The SLIQAS assessed the quality of spine and limb immobilization during transfer of patients to the hospital and consisted of nine items including documentation of the injured limb examination (sensation, movement, distal pulse, and color), immobilization of the joints above and below the affected region, selection of a correct splint size for fixation of fractures, removing the clothing of the injured limbs, no manipulation of the fracture at trauma scene, selection of an appropriate cervical collar (from the shoulder to the chin), keeping the patient’s head in a neutral position (no rotation, flexion, or extension), and aligning the head, trunk, and limbs fixed at a neutral position when the patient was immobilized on the backboard. 

The desirable immobilization was defined as fixation of fractured or sprained region in addition to the joints above and below the affected region. 

The SLIQAS items were scored on a three-point scale in which “two” stood for “Done properly”, “one” for “Done improperly”, and “zero” for either “Not done” or “Not documented”. Accordingly, the total score of SLIQAS ranged from zero to eighteen. Then the total score was divided by nine (the number of questions) to make the criteria for measuring the quality of prehospital immobilization of spine and limbs. Consequently, scores lower and higher than “two” were interpreted as undesirable and desirable quality, respectively. We developed the study questionnaires based on an in-depth literature review. Then, we invited six nursing lecturers to assess the content validity of the questionnaires and their comments were included in the final version of the questionnaires. Content validity index (CVI) was calculated and was equal to “one” meaning that all experts agreed on the relevance of the items (14). In addition, the content validity ratio (CVR) was calculated using Lawsh’es method and it was equal to “one” as all the experts agreed that all the items were essential (15). To ensure the reliability of the instruments, we employed the inter-rater method. Accordingly, two raters administered the study questionnaires to ten patients. The inter-rater correlation coefficient was equal to “one”. Cronbach’s alpha was also calculated using the data from the first 50 questionnaires and was 0.81.

### 3.1. Data Analysis

Data analysis was performed using the Statistical Package for Social Sciences (SPSS v.16.0; SPSS Inc., Chicago, Illinois, USA). There was no missing value. All data were described using frequency tables, central tendency measures, and variability indices. Moreover, Chi-square test was used to assess the association of performing spine and limb immobilization the personnel’s education level, working history, and the type of employment. 

### 3.2. Ethical Considerations

The study protocol was approved by the institutional review board with grant number 9206. 

## 4. Results

**Figure 1.**
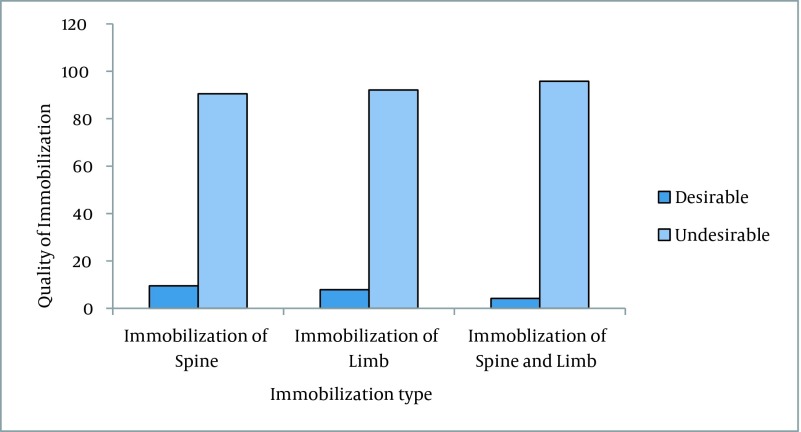
The Quality of Spine and Limb Immobilization in Patients with Multiple Trauma Referred to the Emergency Department

From 400 trauma patients, 301 patients (75.2%) were males. Participants’ age ranged from two to 90 years with a mean age of 34.36 ± 18.59 yrs. Regarding education level, 99 (24.75%) patients were illiterate, 132 (33%) had elementary education, 31 (7.75%) had secondary education, 50 (12.5%) were at high school level, and 67 (16.8%) held diplomas. In addition, 115 (28.75%) patients were workers and among them, 100 (25%) patients were industrial workers (Table 1). Overall, 150 (37.5%) cases of the trauma had occurred during holidays and 261 (65.25%) and 115 (28.75%) cases had occurred in urban streets and on country roads, respectively. The most common mechanism of trauma was RTI in 349 (87.25%) cases. Accordingly, motorcyclists, car passengers, and pedestrians constituted 52.25%, 21%, and 13.25% of the victims, respectively. Moreover, 274 cases of trauma (68.5%) had happened during daytime and 337 (84.25%) of the entire trauma victims had experienced both blunt and penetrating trauma, which were mainly (60%) in the head and neck regions (Table 2). From 228 upper limb injuries, 197 cases had fractures or sprains and 31 (13.59%) cases had superficial injuries (i.e. wound or laceration) without any fracture or sprain. Moreover, from 213 lower limb injuries, 158 cases had fractures or sprains and 55 (25.82%) cases had superficial injuries (i.e. wounded or lacerated) without any fracture or sprain. As Table 3 shows, only 7.88% of the limb immobilizations were at a desirable level of quality while they were undesirable in 92.12% of cases. No significant difference was observed between the desirable and undesirable immobilization in upper and lower limbs (X^2^ = 1.96, P = 0.016). In addition, only 9.5% of the spine immobilizations were in an optimal level of quality. Overall, the quality of immobilization was at undesirable levels in 95.8% of patients with spine or limb injuries and was optimal in only 4.2% of cases (Figure 1). No significant association was observed between the quality of immobilization and work experience (P = 0.13) or the type of employment (P = 0.11) of EMS workers. However, a significant association was observed between the quality of immobilization and the EMS workers’ education level (P = 0.005) (Table 4).

**Table 1.** Participants’ Occupational Status ^a^

Occupational Status	Values
**Self-employment**	94 (23.5)
**Industrial worker**	100 (25)
**Construction worker**	79 (19.75)
**Student**	56 (14)
**Official worker **	15 (3.75)
**Other**	56 (14)

^a^ Data are presented as No. (%).

**Table 2.** Cause of Trauma and the Region of the Injury ^a^

Variable	Values
**Cause of trauma**	
Road traffic injuries	349 (87.25)
Fall	37 (9.25)
Street Fight	10 (2.5)
Falling objects	4 (1)
**Region of injury**	
Head and neck	210 (60.00)
Upper limb	228 (65.14)
Chest	41 (11.71)
Abdomen, back, and pelvis	113 (32.28)
Lower limb	213 (60.85)

^a^ Data are presented as No. (%).

**Table 3.** The Quality of Limb Immobilization in Patients with Limb Fractures ^a^

	Desirable	Done Incorrectly	Not Done
**Involved extremity**			
Upper limb	12 (6.09)	146 (74.12)	39 (19.79)
Lower limb	16 (10.12)	112 (70.89)	30 (18.99)
**Total**	28 (7.88)	258 (72.68)	69 (19.44)

^a^ Data are presented as No. (%).

**Table 4.** The Association between Quality of Spine and Limb Immobilization in Patients with Multiple Trauma with Emergency Medical Services Personnel’s Characteristics ^a^

EMS Characteristics ^b^	Quality of Immobilization		
	Desirable	Undesirable	P Value
**Work History**			0.13
< 5 years	6 (35.3)	210 (54.8)	
5-10 years	4 (23.5)	91 (23.8)	
> 10 years	7 (41.2)	82 (21.4)	
**Type of Employment**			0.11
Permanent	8 (47.1)	91 (23.8)	
By contract	7 (41.2)	164 (42.8)	
By subcontract	1 (5.9)	78 (20.4)	
Mandatory services	1 (5.9)	50 (13.1)	
**Education Level**			0.005
Associate degree	5 (29.4)	242 (63.2)	
Bachelor of science	12 (70.6)	141 (36.8)	

^a^ Data are presented as No. (%).

^b^Abbreviation: EMS, emergency medical services.

## 5. Discussion

The results showed that multiple trauma occurred mostly in males aged 34 to 36 years. This finding was consistent with several previous studies (16-18). It seems that men are predisposed to multiple trauma because most of the motorcyclists, cyclists, and truck drivers are men. In addition, men usually choose hazardous jobs that put them at higher risk for accidents. On the other hand, Kashan is an industrial city and industrial workers make up a majority of its population. Therefore, such people are at high risks for trauma. Moreover, those with elementary education constituted the largest portion of trauma victims in the present study. This finding was consistent with the average education level in the general population. However, it was surprising that the education level of the victims were generally neglected in most of the previous reports (19). For instance, Paravar et al. studied the prehospital trauma care in RTIs in an Iranian population but did not report the education levels of the victims (20). In addition, Bayan et al. investigated the profile of nonfatal injuries due to RTIs in an industrial town in India but did not mention such an important issue (16). Tin Tin et al. also studied injuries to cyclists in New Zealand and did not assess the education level of victims (17). People with low levels of education are usually employed in jobs with greater risks for trauma and injuries and hence, this group should receive more attention.

In the present study, RTI was the most common cause of injury. In addition, such injuries were more common among motorcyclists. Nguyen et al. (21) as well as Lin et al. (18) also reported that RTIs accounted for the main cause of trauma. Moreover, Paravar et al. (20) and Dischinger et al. (22) reported that motorcyclists made up the largest number of patients. However, Engel et al. reported that in Germany and Australia, motorcyclists accounted for only 9.3% of RTIs (23). In addition, falling was the main cause of trauma in two other studies (24, 25). Such differences in studies may be attributed not only to the type of the studied population, but also to cultural factors. Unfortunately, motorcycle use in Iran is prevalent and most of the users belong to lower socioeconomic classes who do not have a driving license and do not use a safety helmet. Thus, an accident can result in serious head injury, long-term disabilities and an increase in healthcare costs. 

In the present study, most RTIs occurred on holidays such as the New Year holidays (Norooz) and in the city streets or on roads outside of the city. This finding was consistent with the reports of Janssen and Burns in Australia (24) as well as Fakharian et al. in Iran (26). These findings signify the necessity of developing effective RTIs prevention strategies during holidays. In the current study, the quality of the spine and limbs immobilization was desirable only in 4.2% of cases. In contrast, the quality of spine immobilizations was undesirable in about 90% of cases. This finding was in contrast with the study of Armstrong et al. who reported that about 92% of vertebral column injuries were immobilized during prehospital transportation (27). Prehospital EMS technicians are responsible for initial care and transporting multiple trauma patients. Careless moving and transporting lead to secondary spinal cord and neurovascular injuries that may negatively affect the patients’ outcome. Proper use of devices such as a long spine board, collars, and limb splints can have a major effect on the quality of life (9). In a systematic review, Ahn et al. studied the prehospital care management of potential SCI patients. They concluded that prehospital EMS technicians should be trained to diagnose pending cervical spinal injuries and immobilize patients suspected of having a SCI to a level similar to the emergency physicians (7). Studies have shown that spine immobilization with a long backboard, cervical collar, and head immobilization between towels or foam wedges provide the most stable biomechanical immobilization (28, 29). It seems that EMS staff retraining, establishment of standard immobilization and transportation protocols, along with strengthening the supervisory system in the EMS, would positively affect the quality of prehospital care, immobilization, and transportation of patients with multiple trauma. 

In the present study, no significant association was found between the quality of spine and limb immobilization and work experience or the type of employment of EMS workers. However, a significant association was observed between the quality of spine and limb immobilization and the EMS workers’ education level. EMS staff with higher qualifications had immobilized the spine and limbs better than the staff with lower qualifications.

The quality of prehospital spine and limb immobilization was undesirable in the majority of patients with multiple trauma transported by the EMS. All data collections and observations in this study were conducted by the second author, which decreased the possibility of inter-rater variations. However, the study was conducted during six months and in only one center. Therefore, the data may not necessarily reflect the performance of all EMS staff nationwide.
